# Effects of *Lactobacillus*-fermented low-protein diets on the growth performance, nitrogen excretion, fecal microbiota and metabolomic profiles of finishing pigs

**DOI:** 10.1038/s41598-024-58832-y

**Published:** 2024-04-14

**Authors:** Hui Liu, Sixin Wang, Meixia Chen, Haifeng Ji, Dongyan Zhang

**Affiliations:** Institute of Animal Science and Veterinary Medicine, Beijing Academy of Agriculture and Forestry Sciences, Beijing, 100097 China

**Keywords:** Biotechnology, Microbiology

## Abstract

This study investigated the effects of *Lactobacillus-*fermented low-protein diet on the growth performance, nitrogen balance, fecal microbiota, and metabolomic profiles of finishing pigs. A total of 90 finishing pigs were assigned to one of three dietary treatments including a normal protein diet (CON) as well as two experimental diets in which a low-protein diet supplemented with 0 (LP) or 1% *Lactobacillus*-fermented low-protein feed (FLP). In comparison with CON, the LP and FLP significantly increased average daily gain (*P* = 0.044), significantly decreased feed to gain ratio (*P* = 0.021), fecal nitrogen (*P* < 0.01), urine nitrogen (*P* < 0.01), and total nitrogen (*P* < 0.01), respectively. The LP group exhibited increased abundances of unclassified_f_Selenomonadaceae, *Coprococcus*, *Faecalibacterium*, and *Butyricicoccus*, while the abundances of Verrucomicrobiae, Verrucomicrobiales, Akkermansiaceae, and *Akkermansia* were enriched in the FLP group. Low-protein diet-induced metabolic changes were enriched in sesquiterpenoid and triterpenoid biosynthesis and *Lactobacillus*-fermented low-protein feed-induced metabolic changes were enriched in phenylpropanoid biosynthesis and arginine biosynthesis. Overall, low-protein diet and *Lactobacillus*-fermented low-protein diet improved the growth performance and reduce nitrogen excretion, possibly via altering the fecal microbiota and metabolites in the finishing pigs. The present study provides novel ideas regarding the application of the low-protein diet and *Lactobacillus*-fermented low-protein diet in swine production.

## Introduction

In modern pig production, the shortage of feed protein sources and environmental pollution caused by excessive nitrogen (N) excretion are of increasing global concern. Feeding pigs with low-protein diets supplemented with individual amino acids (AAs) is an effective strategy for addressing these concerns. According to many studies, reducing dietary crude protein (CP) levels by less than four percentage units, while supplementing some limiting crystalline AAs (mostly lysine, threonine, methionine, and tryptophan) can effectively reduce N excretion without adversely affecting the growth performance of pigs^1,2^. The reductions of dietary CP levels have more significance in finishing pigs for their relatively more consumption of dietary proteins and productions of feces and urine. Recently, branched-chain amino acids (BCAAs), such as leucine, isoleucine, and valine, have demonstrated their potential regulatory effects in nutrition metabolism, gut health, and growth performance^3–5^ and have been suggested to be the next crucial limiting AAs for growing/finishing pigs^6,7^. However, very few studies have investigated the effects of a low-protein diet balanced with 7 essential AAs (lysine, methionine, tryptophan, threonine, and BCAAs) on the growth performance, N excretion, fecal microbiota, and metabolomic profiles of finishing pigs.

*Lactobacillus*, an essential member of the intestinal microbiota, can improve the intestinal microbiota balance and exert beneficial effects on animal hearth^8,9^. In recent years, *Lactobacillus*-fermented feed have been received considerable attention in pig feedstuffs^10,11^. During the microbial fermentation, *Lactobacillus* can utilize nutrients in feed to proliferate and metabolize to produce organic acids, enzymes, extracellular polysaccharides, and other metabolites, which can lower the pH of feed and gut^12^, increase nutrient digestibility^13^, thereby improve performance of pigs^14^. However, reports about the use of *Lactobacillus*-fermented low CP diets in finishing pigs are lacking.

*Lactobacillus paracasei* (*L. paracasei*), a gram-positive, lactic acid-fermenting bacterium, has been used as a strain of probiotic in pig production^15,16^. Here, we used *L. paracasei* ZLP019 to determine the effects of *Lactobacillus*-fermented low-protein diets on the growth performance of and nitrogen balance in finishing pigs. The effects of these diets on fecal microbiota and metabolites in these pigs were also investigated.

## Results

### Growth performance

As shown in Table 1, compared with the normal protein diet group (CON), the low-protein diet group (LP) and *Lactobacillus*-fermented low-protein feed group (FLP) exhibited a higher average daily gain (ADG) (*P* = 0.044) and a lower feed to gain ratio (F/G) (*P* = 0.021), respectively. No differences were observed in the initial body weight (BW), final BW, and average daily feed intake (ADFI) among the three groups.

**Table 1:** Growth performance of finishing pigs fed with *Lactobacillus*-fermented low-protein diets (n = 9).

Items	CON	LP	FLP	SEM	*P* value
Initial body weight, kg	98.7	99.5	99.7	2.91	0.992
Final body weight, kg	124.8	127.3	127.6	3.13	0.941
Average daily weight gain, kg/d	1.00^a^	1.07^b^	1.07^b^	0.02	0.044
Average daily feed intake, kg/d	3.43	3.55	3.50	0.07	0.806
Feed to gain ratio, F/G	3.41^b^	3.32^a^	3.27^a^	0.02	0.021

CON, Normal crude protein diet group; LP, Low crude protein diet group; FLP, *Lactobacillus*-fermented low-protein feed group; SEM, Standard error of means.

^a,b^Means within a row with different superscripts differ significantly (*P* < 0.05).

### Nitrogen balance

As shown in Table 2, compared with the CON group, the LP and FLP groups displayed a lower fecal N (FN), urine N (UN), and total N (TN) (*P* < 0.01), respectively. No differences were observed in N intake (NI), retained N (RN), N retention rate, and N apparent biological value (NABV) among the three groups.

**Table 2:** Nitrogen balance of finishing pigs fed with *Lactobacillus*-fermented low protein diets (n = 9).

Items	CON	LP	FLP	SEM	*P* value
Nitrogen intake, g/d	67.49	59.69	59.86	2.30	0.326
Fecal nitrogen, g/d	12.29^b^	9.57^a^	9.73^a^	0.44	0.001
Urinary nitrogen, g/d	18.36^b^	14.74^a^	14.70^a^	0.62	0.001
Total nitrogen, g/d	30.66^b^	24.31^a^	24.43^a^	1.06	0.001
Retained nitrogen, g/d	36.84	35.38	35.43	1.89	0.952
Nitrogen retention rate, %	54.36	59.04	58.69	1.53	0.418
Nitrogen apparent biological value, %	66.51	70.34	70.15	1.30	0.458

CON, normal crude protein diet group; LP, low crude protein diet group; FLP, *Lactobacillus*-fermented low-protein feed group; SEM, standard error of means.

^a,b^Means within a row with different superscripts differ significantly (*P* < 0.05).

### Fecal microbiota compositions

**Figure 1:**
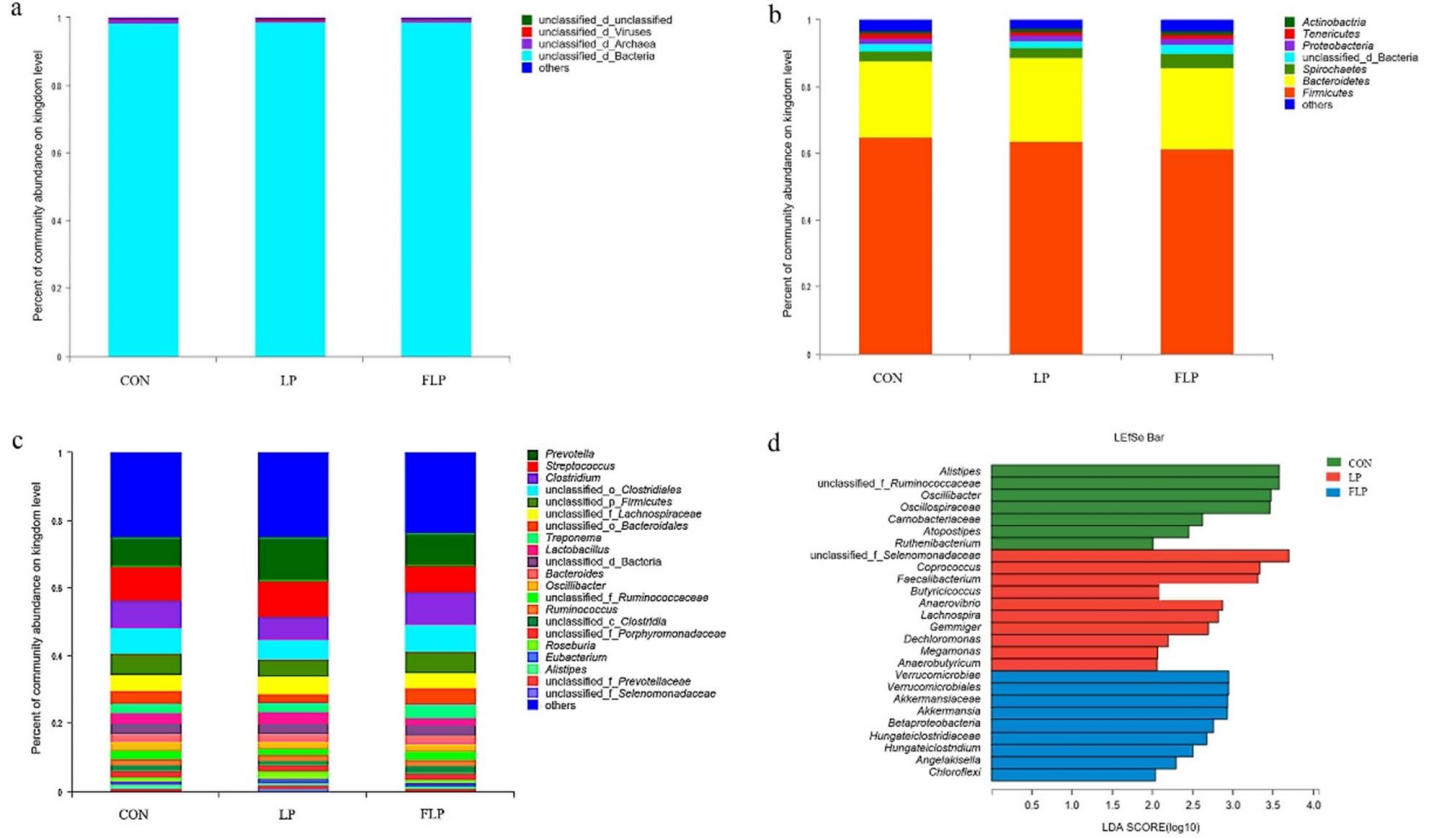
Relative abundances of fecal microbiota in finishing pigs. (**a**) domain; (**b**) phylum; (**c**) genus. (**d**) LEfSe analysis of fecal microbiota, *P* < 0.05 and LDA > 2.0. CON, Normal crude protein diet group; LP, Low crude protein diet group; FLP, *Lactobacillus*-fermented low-protein feed group.

The fecal microbiota compositions at the kingdom, phylum, and genus levels were presented in Fig. 1. At the domain level (Fig. 1a), the four main microbes observed were bacteria (98.69%), archaea (0.66%), viruses (0.40%), and eukaryotes (0.15%). Among the three groups, the highest proportion of bacteria (98.86%) and viruses (0.45%) were observed in the LP group, and the highest proportion of archaea (0.85%) was observed in the CON group. Furthermore, the lowest proportion of eukaryotes (0.12%) was in the CON group. Among the identified bacterial, the four dominant bacterial phyla were Firmicutes (63.23%), Bacteroidetes (24.05%), Spirochaetes (3.44%), and Proteobacteria (1.59%) in all the fecal samples (Fig. 1b). Among the three groups, the relative abundance of Firmicutes (64.82%) was the highest in the FLP group, followed by the CON (63.45%) and LP (61.39%) groups. The abundance of Bacteroidetes (22.84%) in the FLP group was the lowest. The abundance of Bacteroidetes in the LP and CON groups was 24.22% and 25.28%, respectively. The LP group had the highest abundance of Spirochaetes and Proteobacteria (4.35% and 1.84%, respectively), followed by the CON and FLP groups (2.92% and 2.98%; 1.56% and 1.37%, respectively). The relative abundance of the other microbes at the phylum level was < 0.1%. Regarding the domain archaea, the phylum Euryarchaeota was the most dominant member, with the relative abundances of 94.55%, 91.98%, and 88.58% in the CON, LP, and FLP groups, respectively. Moreover, Streptophyta and Uroviricota were the dominant phyla of Eukaryota and Viruses, respectively. At the genus level, microbial abundance varied in different groups, and the six dominant microbes in all the fecal samples were *Prevotella* (10.6%), *Streptococcus* (9.45%), *Clostridium* (8.12%), *Treponema* (3.15%), *Lactobacillus* (3.11%), and *Bacteroides* (2.41%) (Fig. 1c).

Among the three groups, statistical differences in bacterial abundances from the phylum level to the genus level were analyzed using the linear discriminant analysis (LDA) effect size (LEfSe) method (Fig. 1d). The screening conditions were *P* < 0.05 and LDA > 2.0. The abundance of *Alistipe*, unclassified_f_Ruminococcaceae, *Oscillibacter*, Oscillospiraceae, Carnobacteriaceae, *Atopostipes*, and *Ruthenibacterium* were increased in the CON group, while the abundances of unclassified_f_Selenomonadaceae, *Coprococcus*, *Faecalibacterium*, *Butyricicoccus*, *Anaerovibrio*, *Lachnospira*, *Gemmiger*, *Dechloromonas*, *Megamonas*, and *Anaerobutyricum* were increased in LP group. Moreover, the abundances of Verrucomicrobiae, Verrucomicrobiales, Akkermansiaceae, *Akkermansia*, Betaproteobacteria, Hungateiclostridiaceae, *Hungateiclostridium*, *Angelakisella*, and *Chloroflexi* were increased in the FLP group.

### Fecal metabolomic profiles

A total of 730 metabolites were identified in the three groups. Among them, 476 were in the positive ion mode (ESI +), whereas 254 were in the negative ion mode (ESI −). According to the human metabolome database (HMDB), 531 metabolites were mainly annotated to fatty acyls (20.90%), prenol lipids (17.14%), carboxylic acids and derivatives (12.99%), organooxygen compounds (10.89%), and steroids and steroid derivatives (7.63%) in the class term. In the subclass term, 526 metabolites were primarily annotated to amino acids, peptides, and analogues (12.43%), fatty acids and conjugates (9.79%), sesquiterpenoids (4.90%), triterpenoids (3.58%), and carbohydrates and carbohydrate conjugates (3.39%).

**Figure 2:**
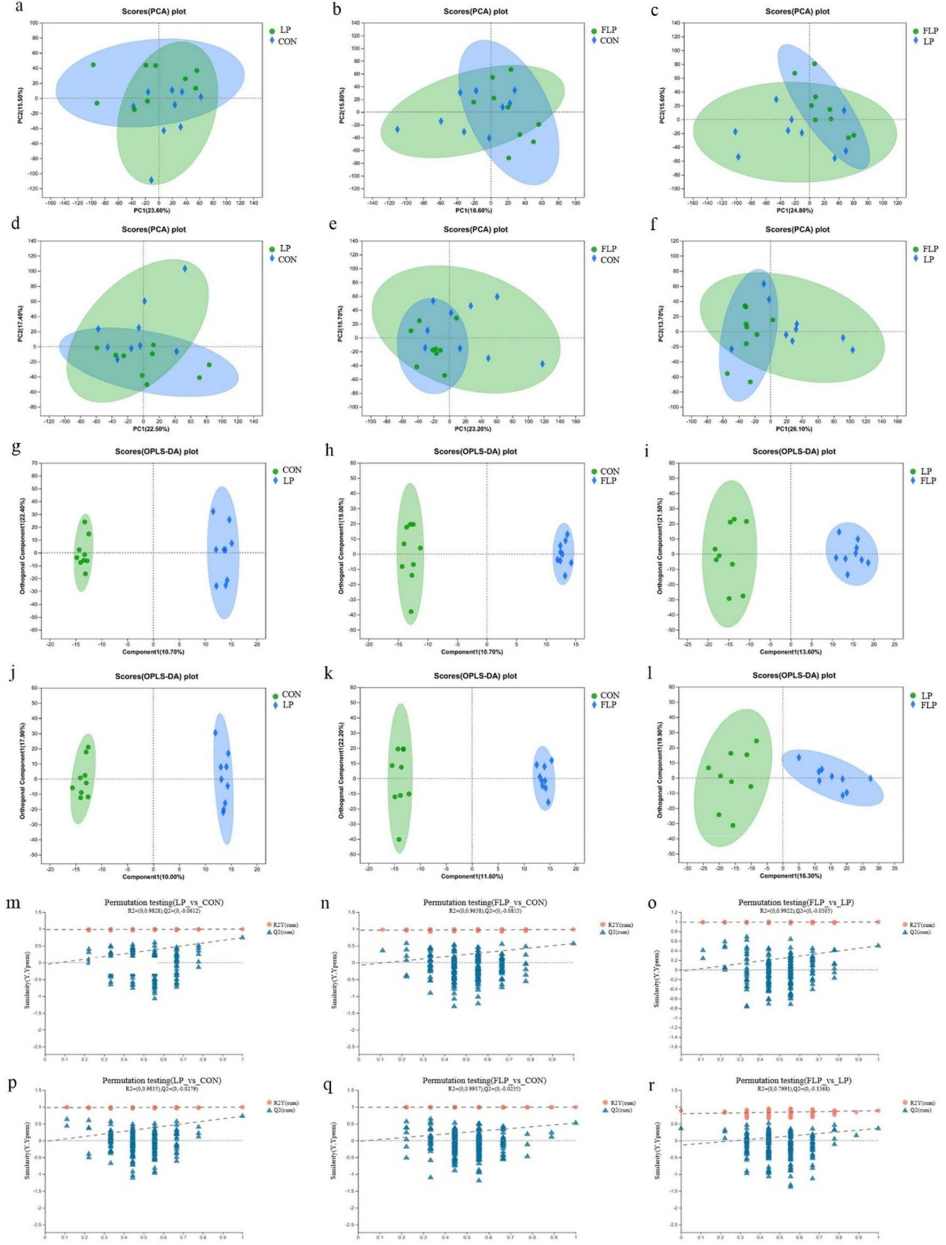
The PCA model score scatter plot (**a**–**f**), OPLS-DA model (**g**–**l**), and permutation tests for the OPLS-DA model (**m**–**r**) of fecal metabolites in the CON, LP, and FLP groups. (**a**–**c**, **g**–**i**, **m**–**o**) were derived from ESI +; (**d**–**f**,** j**–**l, p**–**r**) were derived from ESI −. CON, Normal crude protein diet group; LP, Low crude protein diet group; FLP, *Lactobacillus*-fermented low-protein feed group.

No obvious separation of the principal component analysis (PCA) plot occurred in the fecal metabolites of CON, LP, and FLP groups (Fig. 2a–f). The orthogonal partial least squares discrimination analysis (OPLS-DA) score plots were clearly separated within and between the groups (Fig. 2g–l). The R^2^Y intercepts (> 0.50) and the Q^2^ intercepts (< 0.00) of the permutation test (n = 200) in the OPLS-DA analysis suggested that the model had good adaptability and high predictability in identifying the differential fecal metabolites (Fig. 2m–r).

**Figure 3:**
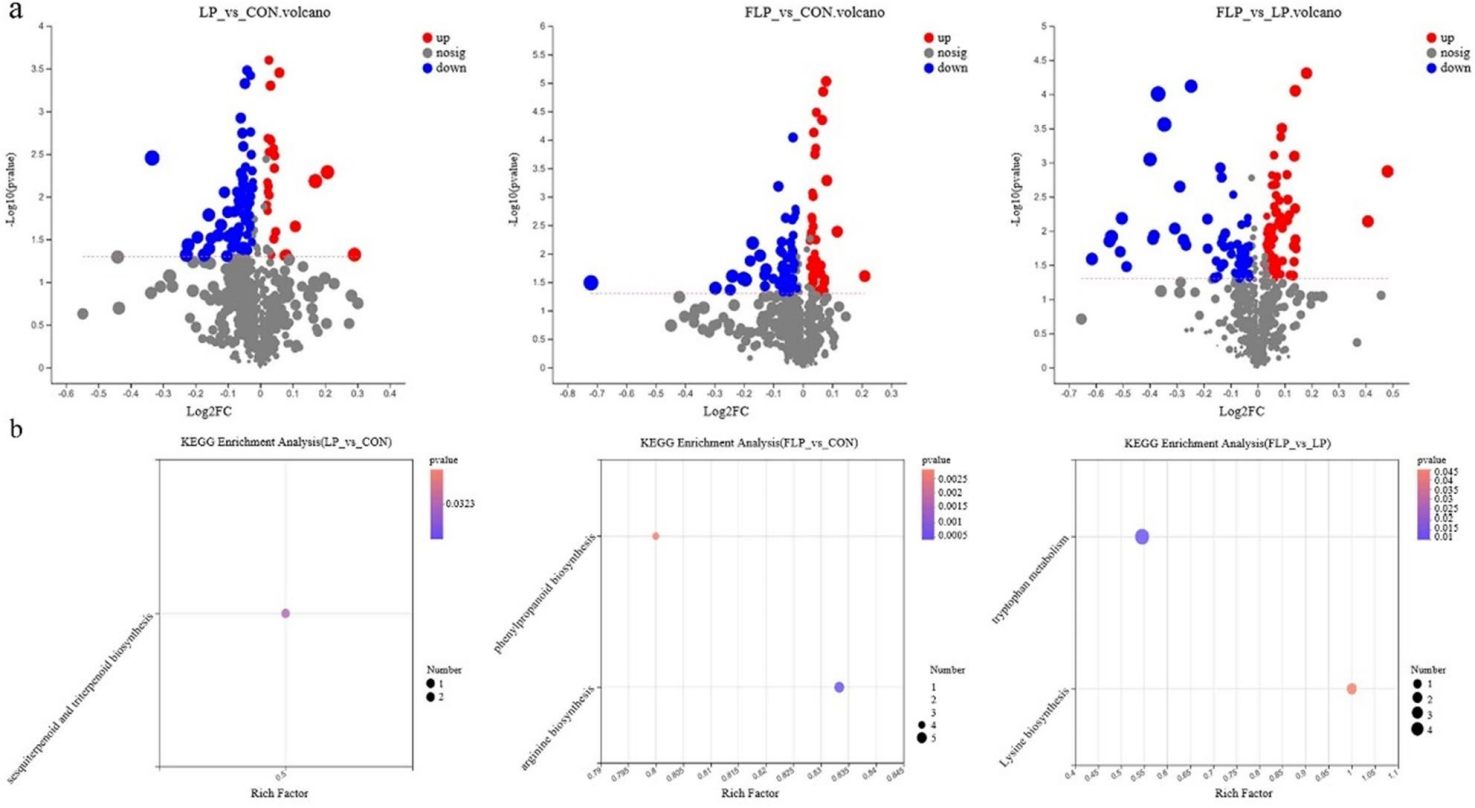
The volcano plot of differential metabolites and the KEGG pathway analysis of significantly changed metabolites. (**a**) The differential metabolites identified from the three comparisons (LP vs. CON, FLP vs. CON, and FLP vs. LP) in ESI + and ESI −. (**b**) KEGG annotations and enrichment of differentially expressed metabolites from the three comparisons (LP vs. CON, FLP vs. CON, and FLP vs. LP). Colored data points in the plots represent statistical significance (*P* < 0.05, VIP ≥ 1.0, and FC > 1.2 or < 0.83). CON, Normal crude protein diet group; LP, Low crude protein diet group; FLP, *Lactobacillus*-fermented low-protein feed group.

Fold changes (FCs) in differential metabolite expression were presented using volcano plots (Fig. 3a). Using a threshold of *P* < 0.05, variable importance in projection (VIP) ≥ 1.0, and FC > 1.2 or < 0.83, the analysis revealed that 19 significant differential metabolites were presented (Table 3). Compared with CON, LP exhibited a downregulation of the relative concentration of L-histidinol, and an upregulation of the relative concentration of 3,4,5-trihydroxy-6-{3-hydroxy-5-[(E)-2-(4-hydroxyphenyl)ethenyl]-2-[(1E)-3-methylbuta-1,3-dien-1-yl]phenoxy}oxane-2-carboxylic acid. FLP exhibited a downregulation of the relative concentrations of Ne,Ne dimethyllysine and spermidine. Compared with LP, FLP exhibited an upregulation of the relative concentrations of 6-[(E)-2-(4-hydroxyphenyl)ethenyl]-4-methoxy-5,6-dihydro-2H-pyran-2-one and L-histidinol, whereas exhibited a downregulation of the relative concentrations of calabaxanthone, 9-hydroxycalabaxanthone, S-acetylphosphopantetheine, (+)-15,16-dihydroxyoctadecanoic acid, (+ /-)-3-[(2-methyl-3-furyl)thio]-2-butanone, PC-M5', 4-(2-aminophenyl)-2,4-dioxobutanoic acid, spermidine, pantetheine, 2-formaminobenzoylacetate, dopaquinone, 4-hydroxy-2-quinolone, 5-hydroxyindoleacetic acid, 4-formyl indole, and serylserine.

**Table 3:** Differential fecal metabolites in finishing pigs.

Metabolites	Subclass	FC	*P* value	VIP	Type
L-Histidinol	Amines	0.79	0.004	3.30	Down
3,4,5-trihydroxy-6-{3-hydroxy-5-[(E)-2-(4-hydroxyphenyl)ethenyl]-2-[(1E)-3-methylbuta-1,3-dien-1-yl]phenoxy}oxane-2-carboxylic acid	Stilbene glycosides	1.22	0.048	2.69	Up
Ne,Ne dimethyllysine	Amino acids, peptides, and analogues	0.61	0.033	3.84	Down
Spermidine	Amines	0.81	0.040	2.82	Down
4-(2-Aminophenyl)-2,4-dioxobutanoic acid	Carbonyl compounds	0.76	0.001	3.90	Down
5-Hydroxyindoleacetic acid	Indolyl carboxylic acids and derivatives	0.81	0.009	2.96	Down
6-[(E)-2-(4-hydroxyphenyl)ethenyl]-4-methoxy-5,6-dihydro-2H-pyran-2-one	–	1.40	0.001	3.14	Up
9-Hydroxycalabaxanthone	1-benzopyrans	0.68	0.014	3.16	Down
Pantetheine	Amino acids, peptides, and analogues	0.77	0.012	2.94	Down
Serylserine	Amino acids, peptides, and analogues	0.83	0.016	2.53	Down
PC-M5'	–	0.71	0.033	2.47	Down
(+)-15,16-Dihydroxyoctadecanoic acid	Fatty acids and conjugates	0.70	0.020	2.63	Down
Calabaxanthone	1-benzopyrans	0.65	0.026	3.19	Down
(+ /-)-3-[(2-methyl-3-furyl)thio]-2-butanone	Aryl thioethers	0.71	0.007	3.23	Down
Dopaquinone	Amino acids, peptides, and analogues	0.82	0.002	2.88	Down
2-Formaminobenzoylacetate	–	0.77	9.87E-05	4.70	Down
L-Histidinol	Amines	1.33	0.007	3.01	Up
S-Acetylphosphopantetheine	–	0.69	0.012	3.75	Down
4-Hydroxy-2-quinolone	–	0.79	0.001	4.34	Down
4-formyl Indole	–	0.83	0.014	3.02	Down
Spermidine	Amines	0.76	0.013	2.97	Down

FC, Fold change; VIP, Variable importance in projection.

Based on the Kyoto Encyclopedia of Genes and Genomes (KEGG) database, all the identified differential metabolites were subjected to the metabolic pathway enrichment analysis (Fig. 3b). The results revealed that the major changed metabolites between the LP and CON groups were enriched at sesquiterpenoid and triterpenoid biosynthesis, the major changed metabolites between the FLP and CON groups were enriched at phenylpropanoid biosynthesis and arginine biosynthesis, and the major changed metabolites between the FLP and LP groups were enriched at tryptophan metabolism and lysine biosynthesis.

### Correlation between fecal microbiota and metabolites

**Figure 4:**
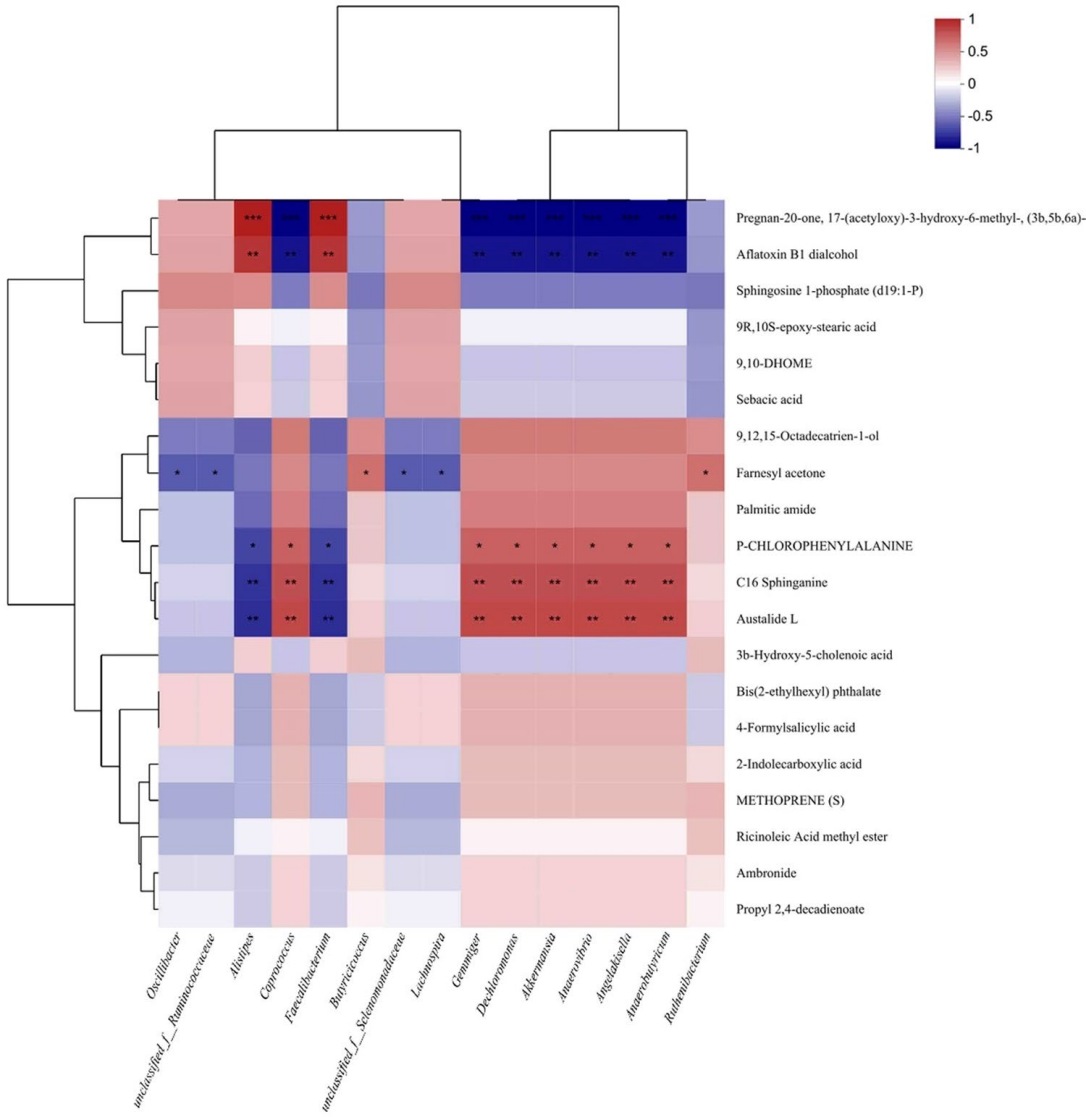
Heatmap of Spearman’s correlation between the abundances of top 15 genera and metabolites. Significant correlations are noted by * for *P* < 0.05, ** for *P* < 0.01, and *** for *P* < 0.001. CON, Normal crude protein diet group; LP, Low crude protein diet group; FLP, *Lactobacillus*-fermented low-protein feed group.

To determine the possible functional relationships between fecal microbiota and fecal metabolites, the Spearman correlation analysis was performed to analyze the correlation between the abundance of top 15 genera and the metabolites. According to the results (Fig. 4), the abundances of *Akkermansia* and *Angelakisella* were positively correlated with pregnan-20-one, 17-(acetyloxy)-3-hydroxy-6-methyl-, (3b,5b,6a)- (*P* < 0.001) and aflatoxin B1 dialcohol (*P* < 0.01), whereas negatively correlated with austalide L (*P* < 0.01), C16 sphingosine (*P* < 0.01), and p-chlorophenylalanine (*P* < 0.05). In addition, the abundances of *Anaerovibrio*, *Coprococcus*, *Faecalibacterium*, *Butyricicoccus*, unclassified_f_Selenomonadaceae, *Lachnospira*, and *Gemmiger* were positively correlated with austalide L (*P* < 0.01), C16 sphingosine (*P* < 0.01), and p-chlorophenylalanine (*P* < 0.05), whereas negatively correlated with pregnan-20-one, 17-(acetyloxy)-3-hydroxy-6-methyl-, (3b,5b,6a)- (*P* < 0.001) and aflatoxin B1 dialcohol (*P* < 0.01). Furthermore, the abundances of *Anaerobutyricum* and *Dechloromonas* were positively correlated with famesyl acetone (*P* < 0.05), as well as the abundances of *Oscillibacter*, unclassified_f_Ruminococcaceae, *Ruthenibacterium*, and *Alistipes* were negatively correlated with famesyl acetone (*P* < 0.05).

## Discussion

In pig production, feed formulators tend to decrease the content of soybean meal to reduce the levels of dietary CP, which may lead to the deficiency of the dietary AA concentrations and poor growth performance^17^. Supplementing low CP diets of pigs with essential AAs can reverse these negative effects, achieving similar performance to normal CP diet-fed pigs^18,19^. Meanwhile, some studies had shown that the performance could be further improved by supplementation of crystalline AAs in pigs fed low CP diets. For example, Jiao et al.^20^ found that supplementing low-protein diets (11.6% CP) with tryptophan, valine, and isoleucine could significantly increase the ADG of finishing pigs, compared with the control CP (15.6%) diet. Li et al.^21^ indicated that low protein diet supplemented with BCAAs (lysine, methionine, threonine and tryptophan) could improve the feed conversion ratio of growing pigs. The present study also demonstrated that reducing the dietary protein levels from 14 to 12% and supplementing the diet with 7 essential AAs could improve the growth performances of finishing pigs, as evidenced by the increase in ADG and decrease in F/G. The reason might be that, compared with pigs fed the normal CP diet, the low CP diets supplemented with these 7 AAs could meet the requirements of essential AAs and support the performance of finishing pigs. However, some other studies had demonstrated that AA-supplemented low-protein diets had no positive effects on the growth performance of pigs during the finishing period. For instance, Fan et al.^22^ reported that finishing pigs fed 13% CP exhibited no significant difference in weight gain compared with those fed 16% CP. However, 10% CP significantly decreased the weight gain compared with 16% CP. These differences might be may be owing to the differences in the concentrations of diet compositions, CP levels, fermented feed, dietary AA ratios, the stage of pigs, and the housing environment. For example, Lordelo et al.^23^ reported that the excess or deficient addition of BCAAs to pigs could not increase the feed intake or growth rate, and so, further investigations are warranted to explore this point. Moreover, the present study also showed that pigs in the FLP group exhibited higher ADG and lower F/G than those in the CON group (*P* < 0.05), and had a numerically lower F/G than those in the LP group (*P* > 0.05). This result thus indicated that the *Lactobacillus*-fermented low CP diets had beneficial effects on the growth efficiency of finishing pigs^24^. The reason might be that the *Lactobacillus*-fermented diet had large numbers of *Lactobacillus* and metabolites, which could lower the pH in the digestive tract, increase the activities of several important digestive enzymes, and hence increase the nutrient digestibility of pigs^25–27^.

Addition of synthetic AAs to low-protein diets could reduce N excretion in pig production^7,28^. The main reason might be because of the improved balance of AAs, increased N deposition rate, and effectively reduced N loss^29^. Kim et al.^30^ reported that this reduction was a result of an increased efficiency of dietary AA utilization for tissue protein synthesis. According to the result of Pfeiffera et al.^31^, the higher the balance of dietary AAs, the less AAs in the liver were used for deamination oxidation, and the less N was excreted. In the present study, a 2% reduction in dietary CP levels balanced with AAs significantly reduced excretion of FN, UN, and TN in the finishing pigs. These results revealed that the low-protein diet (12% CP) and *Lactobacillus*-fermented low CP diet could markedly reduce N excretion in the finishing pigs, similar to previous results^18,32^. Furthermore, the protein level of the diet could affect the amount of emitted Leek et al.^33^ revealed that every 1% reduction in dietary CP supplemented with AAs resulted in a reduction in TN excretion by approximately 8%. Lordelo et al.^23^ also reported a 10% reduction in TN excretion for every 1% reduction in the CP level. Similar results were observed in the present study. In the LP and FLP groups, FN excretion decreased by 22.13% and 20.83%, UN excretion decreased by 19.72% and 19.93%, and TN excretion decreased by 20.71% and 20.32%, respectively (Table 2). These data showed an approximately 9.7% reduction in TN excretion for every 1% reduction in the CP level of the diet supplemented with crystalline AAs. Interestingly, our results suggested that the percentage of reduction in FN excretion exceeded that of UN excretion in the low-protein diet-fed pigs. This might be because the supplemented AAs increased gut AA decarboxylation^34^ or the fermented feed induced changes in gut microbiota fermentation. However, the exact underlying mechanism is unclear and requires to be investigated in future studies.

The gut microbiota is a complex system that could strongly affect pig health through supply of various nutrients, regulation of nutrient metabolism, and modulation of the immune system^35,36^. At the phylum level, the present study revealed that the most abundant populations in the finishing pigs were Firmicutes (63.23%) and Bacteroidetes (24.05%), accounting for over 80% of bacterial communities. The relative abundances of other phyla, such as Spirochaetes (3.44%) and Proteobacteria (1.59%), were present at low abundances (Fig. 1b). This finding was similar to other studies reporting Firmicutes and Bacteroidetes were the primary phyla in the feces of pigs fed with diets with variable protein^18,37,38^. Many studies had indicated that low CP diets supplemented with AAs could alter the intestinal microbiota composition^39,40^. For instance, Zhao et al.^41^ found that the Firmicutes/Bacteroidetes (F/B) ratio markedly reduced when the dietary protein level was reduced by 2% and the diet was balanced with 10 essential AAs (lysine, methionine, threonine, tryptophan, valine, isoleucine, phenylalanine, histidine, leucine and arginine). However, the present results showed that, compared with the CON and LP groups, the FLP group had the highest abundance of Firmicutes (64.82%) and the lowest abundance of Bacteroidetes (22.84%), with the highest F/B ratio (2.84 vs. 2.51 and 2.53, *P* < 0.05). Firmicutes are considered crucial for carbohydrate metabolism as they generate short chain fatty acids (SCFAs)^42^, while Bacteroidetes play a key role in carbohydrate degradation and N metabolism^43^. These findings suggested that the addition of fermented low CP diets could change the composition of microbiota. We speculated that these differences might be attributable to the different diet compositions, dietary CP levels, or the experimental duration, but the exact mechanism need to be studied further.

Based on the LEfSe analysis at genus level, our study revealed that the low CP diet remarkably enhanced the abundance of SCFA-producing bacteria in the LP group, such as *Coprococcus*, *Lachnospira*, *Anaerobutyricum*, *Faecalibacterium*, *Butyricicoccus*, and *Dechloromonas*^44^. *Coprococcus*, *Lachnospira*, and *Anaerobutyricum*, which belong to Lachnospiraceae, can anaerobically convert low-digestible polysaccharides into SCFAs. *Faecalibacterium*, members of the Firmicutes phylum, can produce SCFAs that are beneficial for the host^45^. *Butyricicoccus* and *Dechloromonas*, which belong to Proteobacteria, can utilize volatile fatty acids and aromatic compounds as a carbon source^46^. *Megamonas*, *Anaerovibrio,* and unclassified_f_Selenomonadaceae, which belong to Selenomonadaceae, can synthesize propionic acid through succinic acid decarboxylation^47^. *Gemmiger* belong to Firmicutes and have anti-inflammatory properties^48^. The low-protein diet here mainly improved the abundances of SCFA-producing bacterial genera, which are involved in plant polysaccharide metabolism. Unlike in the LP group, more beneficial endogenous bacteria, such as *Akkermansia*, *Hungateiclostridium*, and *Angelakisella*, were notably enriched in the FLP group. *Akkermansia* can regulate immune functions, produce SCFAs (mainly acetate and propionate), and competitively inhibit other pathogenic bacteria^49^. *Hungateiclostridium* and *Angelakisella* belong to the family Oscillospiraceae, which had been reported to exert a potentially beneficial effect for the production of butyrate^50^. The microbiota compositions between the FLP and LP groups were inconsistent possibly because microbial fermentation could degrade antinutritional factors and macronutrients, provide *Lactobacillus* and their metabolites in the feed, and then modulate the gut microbiota compositions through dietary manipulation^12^. Although the structure of the gut microbiota composition has remained relatively stable in finishing pigs^51^, the present study revealed that the low-protein diet (12% CP) and the fermented low-protein diet could partly modulate the fecal microbiota community of these pigs. These modulations had influenced the nutrient digestion and absorption in pigs, leading to the improved performance and N excretion observed in this study.

One way in which the microbes work is through their produced metabolites. These metabolites serve as nutrients in energy metabolism and as signal molecules between the microbiota and host^52^. In the present study, metabolomic analysis was performed to explore the differential metabolites in the LP and FLP groups. The major upregulated metabolites included 6-[(E)-2-(4-hydroxyphenyl)ethenyl]-4-methoxy-5,6-dihydro-2H-pyran-2-one, L-histidinol, and 3,4,5-trihydroxy-6-{3-hydroxy-5-[(E)-2-(4-hydroxyphenyl)ethenyl]-2-[(1E)-3-methylbuta-1,3-dien-1-yl]phenoxy}oxane-2-carboxylic acid, whereas the major downregulated metabolites were Ne, Ne dimethyllysine, calabaxanthone, 9-hydroxycalabaxanthone, and S-acetylphosphopantetheine. Most of these differential metabolites were annotated to amines and amino acids, peptides, and analogue, which indicated that the added LP and FLP diets mainly promoted protein and amino acids metabolism. Furthermore, the major metabolites that changed between the LP and CON groups were enriched at sesquiterpenoid and triterpenoid biosynthesis (e.g., costunolide and 4-deacetylneosolaniol) (Fig. 3b). These metabolites are involved in various pharmacological activities including anti-inflammation and antioxidation^53^. Compared with the CON group, the major changed metabolites in the FLP group were enriched at phenylpropanoid biosynthesis and arginine biosynthesis (Fig. 3b). The metabolites involved in phenylpropanoid biosynthesis, such as N-acetyl-L-glutamate 5-semialdehyde, L-aspartic acid, N-acetyl-L-glutamic acid, N2-acetyl-L-ornithine, and citrulline, belong to the carboxylic acids and derivatives. They have the potential to scavenge harmful reactive oxygen species^54^. On the other hand, arginine is an intermediate metabolite in the urea and nitric oxide cycles. Arginine metabolism has crucial role in nitrogen distribution and recycling^55^. Moreover, the major metabolites that changed between the FLP and LP groups were enriched at tryptophan metabolism and lysine biosynthesis (Fig. 3b). Tryptophan is essential for protein synthesis, and some products of tryptophan metabolism, such as 2-formaminobenzoylacetate, 5-methoxyindoleacetate, N-acetylisatin, 4-(2-Aminophenyl)-2,4-dioxobutanoic acid, 5-hydroxyindoleacetic acid, and xanthurenic acid, can affect host physiology and contribute to intestinal and systemic homeostasis in numerous ways^56^. Being the first limiting AA in typical swine diets, lysine can affect nutrient metabolism, hormone production, and immunity^57^. The metabolites involved in lysine biosynthesis, such as L-aspartic acid and tetrahydrodipicolinate, were also identified. L-aspartic acid participates in the urea cycle, while tetrahydrodipicolinate is a major intermediate in the lysine biosynthesis pathway, which can be converted from L-aspartic acid.

The relationship between the top 15 genera and metabolites of the finishing pigs was further explored (Fig. 4). According to the heatmap, *Alistipe*, unclassified_f_Ruminococcaceae, *Oscillibacter*, and *Ruthenibacterium* were mainly abundant in the CON group and were negatively correlated with the levels of famesyl acetone, which belongs to an organic compound class known as sesquiterpenoids. However, *Anaerobutyricum* and *Dechloromonas* were abundant in the LP group and were positively correlated with famesyl acetone, probably because the low-protein diets enriched the abundances of these two genera. These two genera are highly involved in sesquiterpenoid and triterpenoid biosynthesis pathway, and so, the LP group contained higher concentrations of crystalline AAs. In addition, *Anaerovibrio*, *Coprococcus*, *Faecalibacterium*, *Butyricicoccus*, unclassified_f_Selenomonadaceae, *Lachnospira*, and *Gemmiger* were presented in the LP group. They exhibited strong positive correlations with austalide L, C16 sphingosine, and p-chlorophenylalanine, and negatively correlations with pregnan-20-one, 17-(acetyloxy)-3-hydroxy-6-methyl-, (3b,5b,6a)- (*P* < 0.001) and aflatoxin B1 dialcohol (*P* < 0.01). Contrarily, *Akkermansia* and *Angelakisella,* present in the FLP group, were positively correlated with pregnan-20-one, 17-(acetyloxy)-3-hydroxy-6-methyl-, (3b,5b,6a)- (*P* < 0.001) and aflatoxin B1 dialcohol (*P* < 0.01), but negatively correlated with austalide L. Austalide L belongs to phenylpropanoids and polyketides and had multiple biological functions, such as antitumor, anti-inflammatory, and antimicrobial^58,59^. C16 sphingosine is a secondary metabolite that belongs to the class of organic nitrogen compounds. P-chlorophenylalanine inhibits tryptophan hydroxylase, a rate-limiting enzyme in 5-HT biosynthesis^60^. Aflatoxin B1 dialcohol is a nontoxic aflatoxin B1 metabolite, relating to the class of organic compounds. Collectively, the results revealed that part of the modified fecal microbiota could regulate gut metabolism through metabolite–host interactions and was highly correlated with the metabolites. Interestingly, the correlation in the FLP group was contrary to that in the LP group, possibly because microbial fermentation could catabolize nutrients of the feed to generate numerous *Lactobacilli* and metabolites, and hence alter the gut microbiota composition, thereby substantially modifying the fecal metabolic profile of finishing pigs. Although further studies are required to investigate the exact mechanism of this correlation, the fermented low CP diets exhibited great potential in improving performance and reducing fecal nitrogen excretion of finishing pigs.

## Conclusion

Overall, a low-protein diet (2% CP level reduction) and a *Lactobacillus*-fermented low-protein diet improved the growth performance, decreased the fecal nitrogen excretion, as well as influenced the fecal microbial composition and metabolomics profiles of finishing pigs. Although further research is warranted, our present study may provide new mechanistic insights regarding the role of *Lactobacillus*-fermented low-protein diets and will benefit its application in pig production.

## Methods

### Ethical statement

The experimental procedures for this study were approved by the Institute of Animal Husbandry and Veterinary Medicine, Beijing Academy of Agriculture and Forestry Sciences, China (Approval No. IHVM11-2304-33). All methods were carried out in accordance with relevant guidelines and regulations. This study is reported in accordance with ARRIVE guidelines (https://arriveguidelines.org).

### *Lactobacillus* strain and feed fermentation

The *L. paracasei* ZLP019 strain, previously isolated from the feces of healthy pig, was identified and preserved by the China General Microbiological Culture Collection Center (Beijing, China) (CGMCC number 11532). The strain was cultured in 250 mL conical flasks containing 100 mL of MRS (de Mann, Rogosa, and Sharpe) broth inoculated with 1 mL of an 18 h culture. The flasks were then incubated at 37 °C for 12 h and the inoculum thus obtained was used for the inoculation of the solid fermented feed. Total viable cell counts of the strain in MRS broth was determined by colony count technique. Briefly, a pre-prepared test sample (0.1 ml) of 10^−6^ and/or 10^−7^ dilution was poured on to sterile Petri plates containing MRS agar medium and then spread uniformly. The plates were then incubated at 37 °C for 48 h. The colonies were counted and the cell density of broth culture was found as 6.0 × 10^9^ (colony-forming units; CFU)/mL. The *Lactobacillus*-fermented feed was solid fermented by the low CP feed supplemented with broth culture of *L. paracasei* ZLP019*.* The fermentation was carried out in one clean sealed plastic bucket with threaded cover. The optimum fermentation parameters were as follows: feed to fermentation broth ratio of 0.8:1 (w/v), fermentation temperature of 37 °C, and fermentation time of 24 h. The viable count for the final fermented feed was 1.13 × 10^9^ CFU/g and its final pH was 4.3.

### Animals and treatment procedures

Ninety Landrace × Large White healthy crossed finishing pigs (initial BW: 99.29 ± 2.91 kg) were randomly assigned to 3 dietary treatment groups based on sex and body weight. Each treatment group had 3 replicate pens with 10 pigs each pen. The pigs in control group (CON) were fed with a normal protein diet containing 14% CP, and the two experimental pigs were fed a low-protein (12% CP) diet containing 0 (LP) or 1% *Lactobacillus*-fermented low-protein feed (FLP), respectively. The diets were formulated to meet or exceed the NRC recommendations^61^ for 100–135 kg BW pigs (Table 4). The low CP diets were balanced with 7 essential AAs (namely, lysine, methionine, tryptophan, threonine, isoleucine, leucine, and valine). Diets were formulated to contain similar digestible energy content and equal standardized ileal digestible contents of essential amino acids. The pre-trial period was 7 days and the formal feeding period was 26 days. The pigs were housed in an environmentally controlled building and allowed ad libitum access to feed and water. They were weighed on day 0 and day 26, and feed intakes of each pen were recorded weekly. ADG, ADFI, and F/G were calculated accordingly.

**Table 4:** Ingredients and nutrient levels of the basal diets.

Ingredients (g/kg)	Normal protein diet	Low protein diet
Corn	68.6	74
Soybean meal	18	12
Wheat bran	10	10
L-Lysine HCL, 780 g/kg	–	0.15
DL-Methionine, 980 g/kg	–	0.09
Threonine, 980 g/kg	–	0.03
Tryptophan, 980 g/kg	–	0.03
Isoleucine, 990 g/kg	–	0.1
Leucine, 990 g/kg	–	0.15
Valine, 980 g/kg	–	0.09
Limestone	1.2	1.2
Dicalcium phosphate	0.8	0.8
Salt	0.4	0.4
Vitamin and mineral premix^a^	1	1
Total	100	100
Nutrient levels		
Digestible energy^b^, MJ/kg	13.61	13.55
Crude protein^c^, %	14.23	12.09
Lysine^c^, %	0.86	0.87
Methionine^c^, %	0.24	0.26
Tryptophan^c^, %	0.18	0.18
Threonine^c^, %	0.51	0.55
Leucine^c^, %	1.21	1.20
Isoleucine^c^, %	0.55	0.54
Valine^c^, %	0.67	0.66

^a^Vitamin and mineral premix provided per kilogram of complete diet (on an air dry basis): Vitamin A, 300,000 IU; Vitamin D_3_, 30,000 IU; Vitamin E, 3850 mg; Vitamin K, 135 mg; Vitamin B_1_, 250 mg; Vitamin B_2_, 650 mg; Vitamin B_6_, 300 mg; Vitamin B_12_, 2.5 mg; nicotinic acid, 2500 mg; pantothenic acid, 1500 mg; biotin, 75 mg; Cu from copper sulphate, 500 mg; Fe from ferrous sulphate monohydrate, 10,000 mg; Zn from zinc oxide, 8000 mg; Mn from manganese oxide, 2000 mg; I from potassium iodate, 50 mg; Se from sodium selenite, 30 mg.

^b^Calculated values.

^c^Measured values.

### Nitrogen balance study

From day 23 to day 26, feces and urine of each pig were collected and weighed twice daily. After all fresh fecal samples were mixed, 500 g of the mixed samples was added with 50 mL of 10% sulfuric acid and mixed thoroughly. Sulfuric acid (9 N) was added to all urine samples for preservation and mixed. Finally, 100 mL of the mixed samples was collected and preserved at − 20 °C for further analysis. The CP contents in diets and fecal and urine samples were analyzed according to the methods of the Association of Official Analytical Chemists^62^. The N balance was calculated as follows: NI = N level in feed × ADG; FN = N level in feces × mean mass of feces; UN = N level in urine × mean volume of urine; TN = FN + UN; RN = NI − FN − UN; N retention rate = RN/NI × 100%; NABV = RN/ (NI − FN) × 100%.

### Metagenome sequencing and annotation

On day 26, five fecal samples were randomly collected from each group. Each fecal sample was snap frozen in liquid nitrogen and stored at − 80 °C for further analysis. Total genomic DNA of each fecal sample (n = 15) was extracted using the E.Z.N.A. Stool DNA (Omega Biotek, Norcross, GA, USA). After the quality was quantified (concentration > 10 ng/μL and A260/A280 > 1.6), DNA samples were fragmented for paired-end library construction and sequenced on an Illumina NovaSeq 6000 platform (Illumina, USA) at Shanghai Majorbio Bio-pharm Technology Co., Ltd (Shanghai, China). Raw sequencing data were quality controlled using fastp (https://github.com/opengene/fastp). The clean reads were aligned using the SOAPaligner (https://github.com/ShujiaHuang/SOAPaligner). Finally, Diamond (https://github.com/bbuchfink/diamond) was used to align a non-redundant gene catalog with the NCBI NR database (https://ftp.ncbi.nlm.nih.gov/blast/db/FASTA/) and the KEGG database (http://www.genome.jp/keeg) for taxonomic and functional annotation.

### LC–MS analysis of fecal samples

A total of 27 fecal samples (n = 9 per group) were used for LC–MS analysis. First, 50 mg of the fecal sample was homogenized and extracted using the methanol–water solution (4:1, v/v). The sample was ground at 60 Hz for 30 min, stored at − 20 °C for 30 min, and centrifuged at 13,000 rpm for 15 min at 4 °C to collect the supernatant for LC–MS analysis. The analysis was conducted on a Thermo UHPLC-Q Exactive HF-X system equipped with an ACQUITY UPLC HSS T3 column (2.1 mm × 100 mm, 1.8 μm, Waters Corp. Milford, MA, USA) kept at 40 °C. The mobile phases A and B consisted of 0.1% formic acid in water: acetonitrile (95:5, v/v) and 0.1% formic acid in acetonitrile: isopropanol: water (47.5:47.5:5, v/v), respectively. The elution gradient program was as follows: 0 − 3.0 min, 5% − 20% B; 3.0 − 9.0 min, 20% − 95% B; 9.0 − 13.0 min, 95% B; 13.0 − 13.1 min, 95% − 5% B; 13.1 − 16.0 min, 5% B. The injection volume of each sample was 2 μL and the flow rate was 400 μL/min. Quality control samples were injected after every 5 samples.

The raw LC–MS data obtained were uploaded to the Majorbio Cloud Platform (https://cloud.majorbio.com) for analysis. Metabolites were identified by searching the HMDB and the KEGG database. The distributions and separations between treatments were examined through the PCA and OPLS-DA. Significant differences in metabolites were identified between the treatments using the VIP from the OPLS-DA analysis and the *P* value of Student’s t-test (VIP ≥ 1 and* P* < 0.05).

### Statistical analysis

The growth performance, nitrogen balance indices, and fecal enzyme activities were analyzed through one-way analysis of variance by using SPSS 25.0 (IBM Corporation, Somers, NY, USA). *P* < 0.05 was considered statistically significant.

## Data Availability

The datasets of metagenome sequence presented during the current study are available in the NCBI sequence read archive under the accession number PRJNA951897 (http://www.ncbi.nlm.nih.gov/bioproject/951897).
